# Validation of a multiplex chip-based assay for the detection of autoantibodies against citrullinated peptides

**DOI:** 10.1186/ar4039

**Published:** 2012-10-01

**Authors:** Monika Hansson, Linda Mathsson, Thomas Schlederer, Lena Israelsson, Per Matsson, Leonor Nogueira, Per-Johan Jakobsson, Karin Lundberg, Vivianne Malmström, Guy Serre, Rikard Holmdahl, Mats Nystrand, Lars Klareskog, Johan Rönnelid

**Affiliations:** 1 Rheumatology Unit, Department of Medicine, Building D2:01, Karolinska Institutet, Stockholm, SE-17176, Sweden; 2 Department of Immunology, Genetics and Pathology, Rudbeck Laboratory C5, Uppsala University, SE-75185, Sweden; 3 Phadia AB, P.O. Box 6460, Uppsala, SE-75137, Sweden; 4 Phadia Multiplexing Diagnostics GmbH, Tech Gate Vienna, Donau-City-Strasse 1 Vienna, AT-1220, Austria; 5 Laboratory of Epidermis Differentiation and Rheumatoid Autoimmunity, UMR 5165, Centre National de la Recherche Scientifique, U 1056 Inserm, Toulouse III University, Hôpital Purpan, Place du Dr Baylac - TSA 40031, 31059 Toulouse cedex 9, France; 6 Medical Inflammation Research, Medical Biophysics and Biochemistry, Scheeles väg 2, Building B2:04, Karolinska Institutet, Stockholm, SE-17176, Sweden

## Abstract

**Introduction:**

Autoantibodies directed against citrullinated proteins/peptides (ACPAs) are highly specific and predictive for the development of rheumatoid arthritis (RA). Different subgroups of RA patients, which have different prognoses and may require different treatments, are characterized by different autoantibody profiles. The objective of this study was to develop a microarray for the detection of multiple RA-associated autoantibodies, initially focusing on responses against citrullinated epitopes on candidate autoantigens in RA.

**Methods:**

The microarray is based on Phadia's ImmunoCAP ISAC system, with which reactivity to more than 100 antigens can be analyzed simultaneously, by using minute serum volumes (< 10 μl). Twelve citrullinated peptides, and the corresponding native arginine-containing control peptides, were immobilized in an arrayed fashion onto a chemically modified glass slide, allowing a three-dimensional layer with high binding capacity. The assay was optimized concerning serum dilution and glass surface, whereas each individual antigen was optimized concerning coupling chemistry, antigen concentration, and selection of spotting buffer. The performance of each peptide in the ImmunoCAP ISAC system was compared with the performance in enzyme-linked immunosorbent assays (ELISAs). Serum from 927 RA patients and 461 healthy controls from a matched case-control study were applied onto reaction sites on glass slides, followed by fluorescent-labeled anti-human immunoglobulin G (IgG) antibody. Fluorescence intensities were detected with a laser scanner, and the results analyzed by using image-analysis software.

**Results:**

Strong correlations between the ImmunoCAP ISAC system and ELISA results were found for individual citrullinated peptides (Spearman ρ typically between 0.75 and 0.90). Reactivity of RA sera with the peptides was seen mainly in the anticyclic citrullinated peptide 2 (CCP2)-positive subset, but some additional reactivity with single citrullinated peptides was seen in the anti-CCP2-negative subset. Adjusting for reactivity against arginine-containing control peptides did not uniformly change the diagnostic performance for antibodies against the individual citrullinated peptides.

**Conclusions:**

The multiplexed array, for detection of autoantibodies against multiple citrullinated epitopes on candidate RA autoantigens, will be of benefit in studies of RA pathogenesis, diagnosis, and potentially as a guide to individualized treatment.

## Introduction

With the discovery of anti-citrullinated protein/peptide antibodies (ACPAs), the interest in autoantibodies has increased during the last decade, from both a diagnostic and a prognostic RA-perspective. In the former American College of Rheumatology (ACR) 1987 classification criteria for rheumatoid arthritis (RA) [1], the presence of rheumatoid factor (RF) accounted for one of seven criteria, of which four should be met for an RA diagnosis. With the introduction of the new 2010 RA classification criteria [2], the impact of autoantibody serology has accordingly increased, and can now contribute to half of the points needed to classify a patient as having RA.

Commercial ACPA tests generally aim to identify collectively as many antibodies against citrullinated epitopes as possible. However, on the peptide level, the ACPA response in RA patients has been shown to be heterogeneous, as different RA patients show reactivity against different citrullinated peptides [3-8]. Although some studies have investigated the impact of having simultaneous ACPA reactivity to different citrullinated peptides (see, for example, [6-10]), such studies have hitherto been performed with multiple parallel enzyme-linked immunosorbent assay (ELISA) tests, an approach that is laborious and can demand sizeable volumes of scarce serum samples (for example, from historical cohorts). Such studies of multiple detailed ACPA specificities have proven informative concerning both the risk for RA development in the context of risk genes [8,11], and the development of risk of arthritis in healthy individuals [6] as well as in arthralgia patients [7].

Most studies on ACPA fine specificity have so far focused on individual antibody responses to epitopes on three citrullinated autoantigens identified in rheumatoid joints: fibrin/fibrinogen [12,13], vimentin [14], and α-enolase [15,16], as well as the skin protein filaggrin, which was used in the early RA-specific tests, before the discovery of the nature of the ACPA response [17,18]. A smaller number of studies have also investigated the response to epitopes on the cartilage-specific type II collagen (CII), another protein that has been found to be citrullinated in RA joints [19]. This protein poses certain demands on the assay used, as the native, noncitrullinated, triple-helical CII molecule in itself is an autoantigen (anti-collagen II antibodies AC2A), with conformational epitopes that differ from the epitopes in the citrullinated counterpart [20]. The murine counterparts of both ACPA and AC2A to the same epitopes have been crystallized and found to be distinct [20,21]. Practical limitations have probably limited the number of ACPA epitopes investigated in parallel in these earlier specificity studies.

Most commercial ACPA tests investigate only the reactivity against citrullinated antigens, and only very few tests take into account the possibility of reactivity against the protein/peptide backbone by simultaneous investigation of reactivity against the arginine-containing noncitrullinated counterpart. Although the ACPA response in RA has been shown to be citrulline specific, a number of studies have shown false ACPA reactivity with concomitant reactivity to arginine-containing control antigens in inflammatory diseases like pulmonary tuberculosis [22,23], hepatitis C infection with cryoglobulinemia [24], and autoimmune hepatitis [25]. During our work with establishment of different ACPA ELISAs, we also found that certain peptide backbones yield increased ELISA reactivity, irrespective of whether the peptide is investigated in its citrullinated or in its native arginine-containing form. Altogether, these findings argue for the usefulness of including arginine-containing control antigens when investigating multiple citrullinated antigens or unknown patient groups. Although such control wells are normally excluded from commercial ELISAs, to increase cost efficiency, they can easily be added to miniaturized systems in a microarrayed fashion. This was recently done in a microarray, allowing the simultaneous evaluation of up to 10 antigens per chip [26].

In this article, we describe the first version of a new microarrayed platform allowing the simultaneous investigation of more than 100 specific antibodies. In the present form, the platform is used for investigation of the ACPA response against 11 previously described citrullinated peptides and their arginine-containing equivalents, as well as one novel peptide, found to be citrullinated in RA synovial membrane and not described before. The peptides represent epitopes from five human autoantigens (fibrinogen, α-enolase, vimentin, collagen type II, and filaggrin), including multiple epitopes from the four antigens (fibrinogen, α-enolase, vimentin, and collagen type II) that have been found to be citrullinated in RA joints. We show that the heterogeneous ACPA response can be investigated with this technique in a serum-saving way. We foresee that this assay can be used in studies on RA patients in both prognostic and diagnostic contexts.

## Materials and methods

### Subjects

For this study, 984 RA patients and 472 healthy controls from the Epidemiological Investigations in Rheumatoid Arthritis (EIRA) case-control study were investigated. EIRA cases have newly diagnosed (within 12 months of first symptoms) RA according to the 1987 ACR classification criteria [1], according to a rheumatologist, and were aged 18 to 70 years at the time of inclusion. As the blood samples were obtained at the first visit to a rheumatologist, no cases were treated with DMARDs or biologics, whereas some cases were treated with NSAIDs and/or low-dose (< 7.5 mg/day) prednisolone. Controls were randomly selected from the Swedish population registry, with matching for sex, age, and residential area. As 57 RA patients and 11 controls showed nonspecific binding in the microarray that rendered their corresponding assay results somewhat uncertain (see later), these samples were excluded from analysis, and the final set included 927 RA patients and 461 healthy controls. Among the 927 RA patients, 401 (43.3%) were positive in the anti-CCP2 ELISA assay (> 25 arbitrary units (AUs)/ml; Immunoscan RA, Eurodiagnostica, Malmö, Sweden), and the remaining RA patients were anti-CCP2 negative. The EIRA samples selected for this methodologic investigation were thus somewhat biased toward ACPA-negative samples, as compared with the entire EIRA study [27].

Serum samples were drawn at the time of inclusion in the EIRA study and thereafter stored frozen at -70°C. All patients and controls had given written informed consent before participating in the study, which had been ethically approved by the regional ethics committee at Karolinska Institutet.

### Citrullinated peptide microarray

Serum samples were analyzed for the presence of ACPA-specific IgG by using a custom-made microarray, based on the ImmunoCAP ISAC system (Phadia Multiplexing Diagnostics GmbH, Vienna, Austria), containing 12 different citrullinated peptides and their native arginine-containing counterparts. All peptides except Fibβ60-74, Fibα621-635, CCP-1/Fil307-324, and citC1 were obtained at ≥ 95% purity from Innovagen AB (Lund, Sweden). The CCP-1/Fil307-324 peptide was obtained from the same source at 80% purity. The triple helical citC1 peptide was synthesized as described in [28], analyzed with mass spectrometry, Western blots and crystallographic analysis, and checked by ELISA by using specific monoclonal antibodies detecting either triple helical or denatured forms of the peptide [21]. Peptides Fibβ60-74, Fibα621-635, and the corresponding arginine control peptides were obtained from NeoMPS (Strasbourg, France) with a purity of > 80%. All antigens were spotted in triplicate on conventional microscopy glass slides with Teflon-masked reaction fields (Marienfeld GmbH, Marienfeld, Germany) coated with an amine-reactive polymer. The investigated ACPAs are detailed in Table 1. The analyses were performed according to the ImmunoCAP ISAC standard assay procedure [29,30], with some deviations. In brief, samples were diluted 1:40 in dilution buffer (Phadia), and 25 μl was added to the reaction wells of the microarray. After a 2-hour incubation at room temperature in a humidity chamber, the slides were washed, and 25 μl of the secondary antibody, Cy3 conjugated goat anti-human IgG (Jackson ImmunoResearch Laboratories Inc., Newmarket, UK), was added to the reaction sites at a concentration of 1 μg/ml. The slides were incubated for 30 minutes and then washed again before they were scanned by using a laser scanner (LuxScan 10K; CapitalBio, Beijing, China). Fluorescence intensities were recorded, and data processing was performed by using the Microarray Image Analysis software (Phadia). This software calculates intensity homogeneity as a coefficient of variation (CV%) within individual spots, and intraspot CV% within triplicate spots coated with the same peptide. Both these CV% values must be < 25; otherwise, aberrant spots are excluded. At least two spots in a triplicate must be accepted to obtain results.

**Table 1:** Citrullinated peptides in the ACPA microarray

Peptide	Protein	Amino acids	Amino acid sequence	Reference
CEP-1/Eno5-21	α-Enolase	5-21	CKIHA(cit)EIFDS(cit)GNPTVEC (cyclic)	[16]

Vim60-75	Vimentin	60-75	VYAT(cit)SSAV(cit)L(cit)SSVP	[37]

Vim2-17	Vimentin	2-17	ST(cit)SVSSSSY(cit)(cit)MFGG	[33]

CCP-1/Fil307-324	Filaggrin	307-324	SHQEST(cit)GRSRGRSGRSGS (cyclic)	[3,32]

Fibβ36-52	Fibrinogen β-chain	36-52	NEEGFFSA(cit)GHRPLDKK	[37]

Fibβ563-583	Fibrinogen β-chain	563-583	HHPGIAEFPS(cit)GKSSSYSKQF	[13]

Fibβ580-600	Fibrinogen β-chain	580-600	SKQFTSSTSYN(cit)GDSTFESKS	^c^

Fibβ62-81a^a^	Fibrinogen β-chain	62-81	APPPISGGGY(cit)ARPAKAAAT	[13]

Fibβ62-81b^b^	Fibrinogen β-chain	62-81	APPPISGGGYRA(cit)PAKAAAT	[13]

Fibβ60-74	Fibrinogen β-chain	60-74	(cit)PAPPPISGGGY(cit)A(cit)	[12,31]

Fibα621-635	Fibrinogen α-chain	621-635	(cit)GHAKS(cit)PV(cit)GIHTS	[12,31]

citC1/CII359-369	Collagen type II	359-369	(GPO)5-GA(cit)GLTG(cit)PGDA(GPO)2-GKKYG	[20,28]

The arginine-containing control peptides have identical sequences, except that they contain arginine residues instead of citrulline (Cit, citrulline). Peptides are designated by their abbreviated protein names (Eno, α-enolase; Vim, vimentin; Fib, fibrinogen; CII, type II collagen) followed by the positions of the first and last amino acid. For three peptides, earlier given specific names (CEP-1, CCP-1, citC1), these names are given in parallel. ^a^Citrullinated at position 72. Uses the same control peptide containing two arginines as for Fibβ62-81b. ^b^Citrullinated at position 74. Uses the same control peptide containing two arginines as for Fibβ62-81a. ^c^The citrullinated epitope in this peptide has been found to be citrullinated in RA synovial tissue (Per-Johan Jakobsson, personal communication).

### Validation against ELISAs for the individual citrullinated peptides

The performance of the microarray was investigated by comparison with individual ELISAs for the 12 included citrullinated peptides. For each peptide, suitable serum samples (between 40 and 100 samples per peptide) encompassing a broad range of specific reactivities against the individual citrullinated peptide were defined by specific ELISAs, whereupon the same samples were investigated with the microarray. ELISAs for 10 of the peptides were performed at the Department of Rheumatology, Karolinska Institutet, as described in [5]. Eight ELISAs used individual standard curves produced by serum samples with high levels of ACPA of the relevant specificity, and ACPA levels were expressed as arbitrary units (AUs) per milliliter. No corrections for reactivity against arginine control peptides were done in ELISA or microarray data for the corresponding eight peptides. For four of the peptides (Fibβ60-74, Fibα621-635, Fibβ62-81a, and Fibβ62-81b), ELISA results were expressed as optical densities (ODs) after subtraction of the OD for the corresponding arginine-containing peptides, and comparisons were done with the corresponding arginine-subtracted fluorescence intensities from the microarray. ELISAs for the peptides Fibβ60-74 and Fibα621-635 were performed in the laboratory of Professor Guy Serre in Toulouse, according to [31].

### Statistics

Analyses were performed in two ways for each separate citrullinated peptide. In a first investigation, gross data for the results for the 12 citrullinated peptides in the 927 RA patients and 461 healthy controls were compared. In the second evaluation, net ACPA results were calculated by subtracting the fluorescence intensity for the arginine-containing control peptide from each citrullinated peptide for all RA patients and controls, whereupon these net values were used to construct ROC curves and to compare performance. This calculation of net ACPA reactivity was performed for all peptides, except for citCI, for which only uncorrected values were used, as the argC1 peptide is an autoantigen in its own right, and with conformational epitopes differing from the citC1 peptide [20,21].

To allow stringent comparison between performances for the different citrullinated peptides, the cut-off point for each investigation was defined as the fluorescence intensity corresponding to a diagnostic specificity of 98.0%, calculated from the fluorescence intensities of the investigations for the 461 healthy controls.

ACPA responses are known to show a bimodal distribution clearly separating ACPA-positive and ACPA-negative subjects, and studies on the correlation between microarray and ELISA results were therefore performed with the nonparametric Spearman Rank Test.

Receiver operator characteristic (ROC) curves were constructed by using the Analyze-it software (Analyze-it Software Ltd, Leeds, UK), and correlation between ELISA and microarray results was calculated by using the JMP software (SAS Inc., Cary, NC, USA).

## Results

### Performance of the ACPA microarray

**Figure 1:**
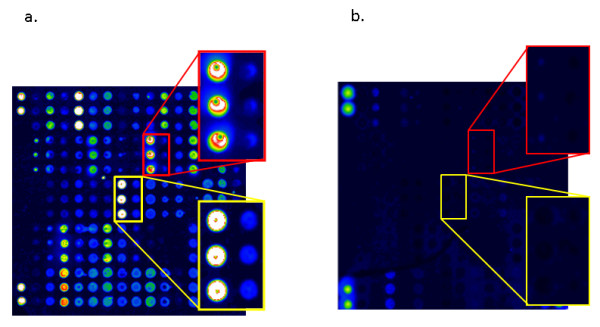
**Graphic representation of the fluorescence intensities obtained from (a) an ACPA-positive RA serum and (b) an ACPA-negative control serum**. Magnifications are shown to the right. The upper red frame shows reactivity to Fibβ60-74, and the lower yellow frame shows reactivity to CEP1/Eno5-21. Within each frame, the left triplicate shows reactivity to the citrullinated peptide, whereas the right triplicate shows the reactivity to the native arginine-containing counterpart. Duplicate spots in the corners of both chips represent non-antigen-dependent fluorescence used for technical alignment of the chips.

When the laser-scanned slides were processed with the Microarray Image Analysis software, the sites where the citrullinated peptides had been spotted in triplicate were clearly visualized after treatment with serum positive for the corresponding autoantibodies. Examples of a positive RA serum and a negative control serum are shown in Figure 1.

**Figure 2:**
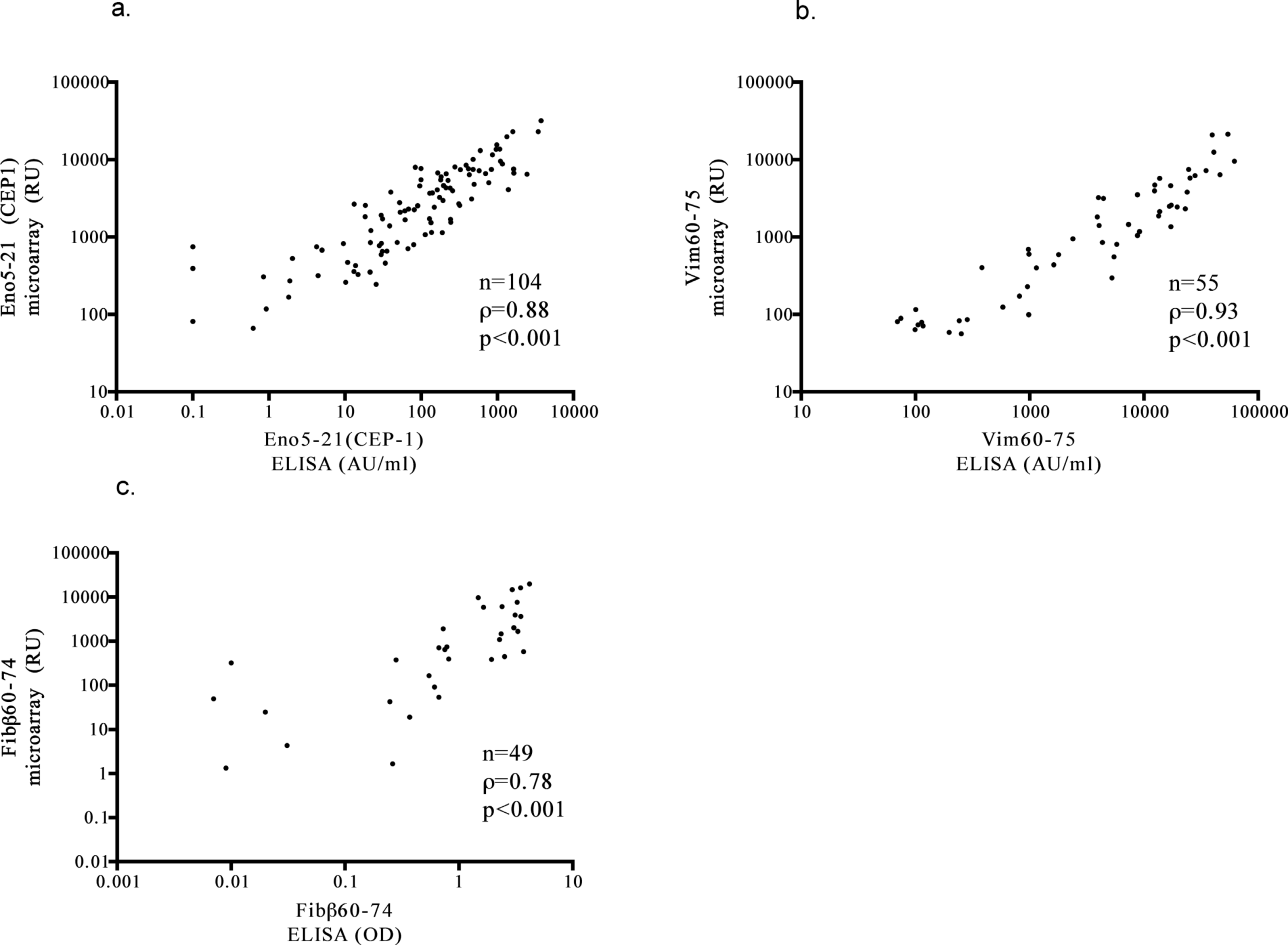
**Correlation between measurements with ELISA and microarray for (a) Eno5-21, (b) Vim60-75, and (c) Fibβ60-74**. In a and b, comparisons were made for uncorrected values, and in c, net values were determined after correction for reactivities against the arginine-containing control peptide.

Comparison of the fluorescence intensities in response units (RUs) with corresponding ELISA units yielded satisfactory correlations between the two techniques, with Spearman ρ values typically between 0.75 and 0.90. Three representative examples are shown in Figure 2a through c.

The inter- and intraassay coefficient of variation (CV%) was investigated by using serum samples with high (15,000 to 60,000 RU), intermediate (1,500 to 15,000 RU), and low (50 to 1,500 RU) levels of antigen-specific antibodies targeting representative citrullinated peptides and their arginine-containing counterparts. The inter- and intraassay CV% for samples with high levels of specific antibodies ranged from 8.3 to 16.8 and 5.3 to 17.0, respectively. For the intermediate samples, the CV% was 9.5 to 22.8 and 10.3 to 19.3, respectively. The corresponding figures for low-level samples were 5.0 to 22 and 13.3 to 31.4.

Besides the difference in reactivity between RA patients (especially the anti-CCP2-positive patients) and the healthy controls, a difference also was found in the size of signals against the individual peptide backbones that was common to both citrullinated peptides and to arginine-containing control peptides, as well as to both RA patients and controls. This fact manifested itself in different cut-off values for the individual peptides: with the highest values for Fibβ580-600 (3,454 RU) and Vim60-75 (2,500 RU) and the lowest for Vim2-17 (256 RU) and Fibβ36-52 (222 RU). Peptides with generally high fluorescence intensities in the microarray also showed generally high OD levels in the parallel ELISA investigations (data not shown).

Staining of the slides with some serum samples yielded a fluorescence signal also from the glass surface surrounding the triplicate areas with spotted antigen. This happened in 57 (5.1%) of 984 of the RA sera and in 11 (2.3%) 472 of the control sera. As this phenomenon of nonspecific binding might conceal weakly reactive spots, we decided to exclude samples with this reactivity pattern from our further analyses. Exclusion of samples with unspecific binding had, however, very limited effect on the performance at group level and on the appearance of ROC curves for the individual citrullinated peptides (data not shown).

### Discriminatory properties of individual citrullinated peptides

**Figure 3:**
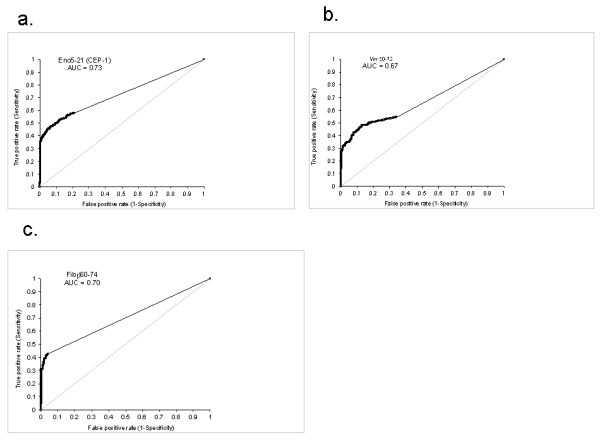
**Receiver operating characteristic (ROC) curves for (a) Eno5-21, (b) Vim60-75, and (c) Fibβ60-74**. The analyses were based on comparisons between 927 RA patients (43.3% anti-CCP2 positive) and 461 healthy controls. AUC, Area under the curve.

When ROC curves were constructed for the individual citrullinated peptides, they generally showed a high specificity pattern, with alignment with the Y axis, as is also seen for anti-CCP2 [32]. Three examples of peptide ROC curves are shown in Figure 3a through c. Notably, substantial differences were noted in the frequency of patients with positive reactivity for the different citrullinated peptides. When the cut-off was set to exclude 98% of the healthy controls for each peptide, the microarray defined between 10.5% (Fibβ580-600) and 45.2% (Fibβ36-52) of the RA patients in the selected EIRA cohort as ACPA positive (Table 2).

**Table 2:** Performance of the 12 investigated citrullinated peptides

Peptide	Percentage of RA sera reacting with the individual citrullinated peptides^a ^(%) at 98.0% specificity	Number of positive patients/number of CCP2-positive patients (%)^a^	Percentage of RA sera reacting with the individual citrullinated peptides (%) at 98.0% specificity after arginine subtraction^b^	Number of positive patients/number of CCP2-positive patients (%)^b ^after arginine subtraction	Positive (+) or negative (-) effect of arginine subtraction on the diagnostic performance
Eno5-21 (CEP-1)	39.4	91.0	40.9	94.5	+

Vim60-75	31.9	73.7	40.0	92.4	+

Vim2-17	10.8	24.9	8.0	18.5	-

Fil307-324 (CCP1)	32.8	75.8	32.5	75.1	-

Fibβ36-52	45.2	104.4	41.9	96.8	-

Fibβ563-583	31.0	71.6	31.5	72.7	+

Fibβ580-600	10.5	24.2	9.8	22.6	-

Fibβ62-81a	19.8	45.7	20.6	47.6	+

Fibβ62-81b	28.0	64.7	26.1	60.3	-

Fibβ60-74	38.8	89.6	37.3	86.1	-

Fibα621-635	25.9	59.8	29.0	67.0	+

CII 359-369 (CitC1)	18.8	43.4	ND^c^	ND^c^	ND^c^

Peptides names are designated as in Table 1.

^a^These percentages should be compared with the fact that 43.3% of all RA patients in the present selection of EIRA patients were anti-CCP2 positive.

^b^Fluorescence intensities for the arginine control peptides were subtracted from the fluorescence intensities for each measurement among RA patients and controls, whereupon the performance was calculated on the net values.

^c^No subtraction was done for this peptide as the arginine control peptide is itself an autoantigen with conformational epitopes that are destroyed by citrullination [20].

### Effects of the subtraction of arginine control reactivities

In parallel to evaluating the diagnostic properties of the crude fluorescence intensities against the citrullinated peptides, we also performed the same evaluation after the fluorescence intensities for the control peptides containing arginine had been subtracted for all samples from RA patients and controls. In some cases, this correction increased the number of RA cases that were positive in the assay (positive reaction defined as values above the threshold set by the 98% negative reaction rates among healthy controls): for reactivity against Vim60-75, the number of positive patients increased from 31.9% to 40.0%, and for Fibα621-635, a corresponding increase was found from 26.9% to 29.0%. For some peptides, subtraction of the arginine control values instead decreased the number of positive sera: for Vim2-17, from 10.8% to 8.0%, and for Fibβ36-52, from 45.2% to 41.9%. For the other investigated peptides, the corresponding differences were smaller, and of the 11 peptides investigated, five showed increased, and six showed decreased frequencies of positive reactions among the RA patients after arginine correction. The performance data for all investigated citrullinated peptides are shown in Table 2.

### Number of peptide reactivities in CCP2-positive and CCP2-negative RA patients

Among the 927 investigated RA patients, the median number of citrullinated peptide reactivities against the 12 investigated peptides was 2, and the mean, 3.33.

**Figure 4:**
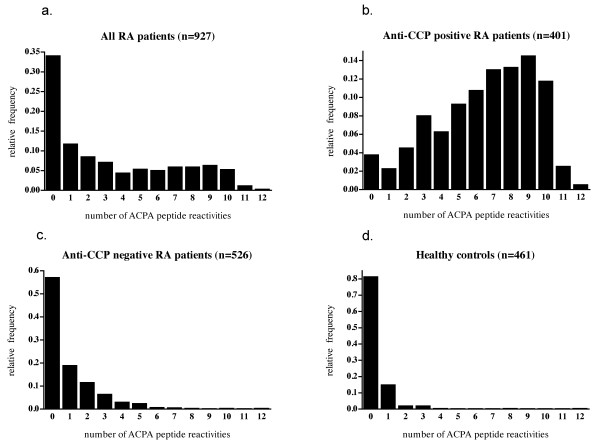
**Histograms showing the relative frequency of subjects as a function of number of peptide reactivities against the 12 investigated citrullinated peptides for (a) all RA patients, (b) CCP-positive RA patients, (c) CCP-negative RA patients, and (d) healthy controls**.

When the patients were split according to anti-CCP2 status, a marked difference between the groups was evident, as the corresponding figures were 7.0 and 6.48 for the anti-CCP2-positive patients and 0.0 and 0.93 for the anti-CCP2-negative patients. The median number of positive reactions among the healthy controls was 0.0 (mean, 0.26). The distributions of the number of positive ACPA reactions in the different groups are graphically shown in Figure 4a through d.

## Discussion

In this report, we describe a microarray in which specific reactivities against multiple citrullinated peptides can be investigated in parallel. Notwithstanding the microarray approach, each one of the individual autoantibody reactivities showed good correlation to results obtained with parallel ELISAs for the individual peptides. With a large validation cohort of RA patients and controls, followed by ROC curve analyses, we could show that the individual analyses showed high diagnostic specificity. At a defined specificity level (98%), the individual peptides showed marked differences in diagnostic performance, with some individual peptides performing similarly to the anti-CCP2 assay.

When the RA patients were split into those positive and negative for anti-CCP2, a large difference was shown in the number of peptide reactivities detected in the two groups. This difference was particularly pronounced concerning individuals with multiple reactivities. However, a few patients also in the anti-CCP2-negative group showed multiple reactivities against the citrullinated peptides in the multiplex assay. This indicates that the microarray might be useful for analyses of residual ACPA reactivities that are not covered by the conventional ACPA tests like anti-CCP2. The value of this assay to generate hypotheses in a diagnostic setting remains to be evaluated by using larger sets of sera from different cohorts of RA patients and disease controls (patients with infections and other noninfectious inflammatory diseases). Of special interest will be the use of the assay to estimate the risk of RA development in patients with early undifferentiated arthritis [6] and in other individuals at risk for RA. In such future studies, it will also be possible to evaluate further the eventual diagnostic value of the individual peptides included in the present multiplex assay.

In the current design, the microarray uses very small quantities of serum (< 10 μl). This is an advantage when available serum volumes are small and when replacement samples are not available (for example, when samples have been retrieved from historic biobanks, containing samples taken from individuals at a time point before the development of RA). We are currently performing such studies on the development of individual ACPA responses in sera from RA patients retrieved from historic biobanks from the years preceding the development of RA. In such studies, an imperative demand exists to use small serum quantities very efficiently.

Certain sera yielded high background reactivities that might obscure the readout for the individual antigens. However, this is not unique to this specific microarray. It is well known, among developers of addressable laser bead immunoassays (ALBIA) and ELISA assays, that certain sera show unspecific binding. Given the setup of most commercial ELISAs, in which each serum is investigated only in wells coated with the antigen in question but without serum-specific control wells, this unspecific binding will often pass unperceived. Because we can readily assess the overall nonspecific binding in the used assay, we have excluded sera with apparent unspecific binding, but have also noticed that inclusion of such sera had very little effect on the performance at group level of the citrullinated peptides used in this investigation.

We defined the cutoff levels as the 98^th ^percentile for the healthy controls, implying that 2% of the healthy controls showed a positive response to each individual peptide. The discriminatory property of each individual citrullinated peptide in this multiplex assay is thus consistent with that of the anti-CCP2 assay when comparing RA patients and healthy controls. For stochastic reasons, this results in a higher number of healthy controls having a positive signal for any of the 12 analyzed peptides. These positive signals were, however, mostly only slightly above the threshold, and in only a very few of the healthy controls was multiple reactivity seen, as was the case in most anti-CCP2-positive RA patients.

Our initial pilot studies indicated a gain in diagnostic sensitivity when subtracting the fluorescence intensities from the arginine control peptides. However, in this validation, by using a large set of RA patients and controls, this was not a universal finding, and similar numbers of peptides showed decrease or increase in diagnostic performance after correction for reactivity to control peptides. We conclude that the issue of arginine subtraction still is an open question, and that the most-suitable approach has to be decided for each antigen/peptide. This is also reflected by the different approaches used in currently available commercial ACPA tests, in which some assays, like the anti-Sa assay from Euroimmun, include sample-specific control wells with noncitrullinated antigen, whereas most assays, like the anti-CCP2 test, do not.

In a recent study by Chandra *et al. *[26], another multiplex approach was described for the detection of ACPAs, by using a chip design with up to 10 antigens per chip. Contrary to our study, biotinylated peptides were used consistently by Chandra *et al.*, and a different setup of peptides was investigated. Thus, several features differ between the two multiplex approaches, in which our method may have certain advantages in allowing more antigens to be investigated in parallel (up to 170 per chip). However, a straight head-to-head comparison of the two approaches to multiplexing will be needed to harmonize the interpretation of ACPA multiplex assaying.

As in our present investigation, the authors of the previous report [26] used no standard curve for normalization of the responses against citrullinated peptides. Given the many different reactivities that are investigated simultaneously, the construction of such a standard is a complex task, and its resolution is not included in the present first methods description of the current platform. However, this issue also addresses future development of our multiplex platform.

A particular issue in any effort to measure antibodies against several different epitopes on citrullinated autoantigens is the degree of cross-reactivity between these different epitopes. Previous ELISA-based investigations have addressed this issue by peptide-absorption experiments and concluded that a large fraction of the antibodies against epitopes included in our assay are not cross-reactive, whereas a smaller fraction of antibodies cross-react between different epitopes [7,8,33]. A further complication is that half of the peptides used in the present study, as well as the seven citrullinated peptides described in the study by Chandra *et al. *[26], have multiple citrullination sites. As shown in Table 2 with the 98% specificity described for anti-CCP2 [32], reactivities against several of the multicitrullinated peptides show a similar degree of positivity as the anti-CCP2 assay does in the same patient group, suggesting the presence of multiple antibody-binding sites. Thus, we cannot rule out the possibility that ACPAs with different fine specificities bind to different epitopes on these particular peptides [31]. Further detailed studies using additional peptides and different absorption approaches as done, for example, in [8], will be needed to resolve these issues.

RA subgroups with different clinical prognosis are characterized by different autoantibody profiles. Besides the ACPA (and RF)-associated RA phenotype associated with poor long-term prognosis, we have, for example, defined an acute-onset RA phenotype associated with high levels of antibodies against native human CII [34,35], whereas others have described a mild RA phenotype associated with antibodies against the RA33 antigen [36]. Our aim is to use the microarray for the simultaneous investigation of such autoantibody-defined RA phenotypes in parallel.

Our long-term aim for the currently described multiplex assay platform is further to expand the number of peptides and protein fragments included, and further to validate the current and future antibody targets in additional clinical cohorts. Such extensions of numbers of targets will be feasible, as abundant positions are left for antigen spotting.

## Conclusions

We have developed a microarray for the simultaneous measurement of multiple specific ACPA responses. The individual analytes have been quantitatively validated against specific ELISAs and show a high specificity comparable to that of the anti-CCP2 assay. We foresee that this assay will have a broad range of applications in studies of prediction, diagnosis, and prognosis of arthritis, as well as in studies of specific ACPA responses before and during conventional and experimental therapies in RA.

## Abbreviations

ACPA: anti-citrullinated proteins/peptide antibody; ACR: American College of Rheumatology; ALBIA: addressable laser bead immunoAssay; AU: arbitrary unit; CCP: cyclic citrullinated peptide; CCP-1: cyclic citrullinated filaggrin peptide encompassing amino acids 307 through 324 (Fil307-324); CEP-1: cyclic citrullinated α-enolase peptide encompassing amino acids 5 through 21 (Eno5-21); citC1: collagen type II peptide encompassing amino acids 359 through 369; CII: type II collagen; CII359-369: collagen type II peptide encompassing amino acids 359-369; CV: coefficient of variation; DMARD: disease-modifying anti-rheumatic drug; EIRA: Epidemiological Investigations in Rheumatoid Arthritis; ELISA: enzyme-linked immunosorbent assay; Eno5-21: cyclic citrullinated α-enolase peptide encompassing amino acids 5-21 (CEP-1); ERC: European Research Council; Fibα621-635: fibrinogen α-chain peptide encompassing amino acids 621-635; Fibβ36-52: fibrinogen β-chain peptide encompassing amino acids 36-52; Fibβ563-583: fibrinogen β-chain peptide encompassing amino acids 563-583; Fibβ580-600: fibrinogen β-chain peptide encompassing amino acids 580-600; Fibβ60-74: fibrinogen β-chain peptide encompassing amino acids 60-74; Fibβ62-81a: fibrinogen β-chain peptide encompassing amino acids 62-81, citrullinated at position 72; Fibβ62-81b: fibrinogen β-chain peptide encompassing amino acids 62-81 citrullinated at position 74; Fil307-324: cyclic citrullinated filaggrin peptide encompassing amino acids 307-324 (CCP1); IMI: Innovative Medicines Initiative; ISAC: immuno solid phase antigen chip; NSAID: nonsteroidal antiinflammatory drug; OD: optical density; RA: rheumatoid arthritis; RF: rheumatoid factor; ROC: receiver operating characteristic; RU: response unit; Vim60-75: vimentin peptide encompassing amino acids 60-75; Vim2-17: vimentin peptide encompassing amino acids 2-17.

## Competing interests

The project is part of the Innovative Medicines Initiative (IMI) project BeTheCure, in which Karolinska Institutet is a scientific partner and Phadia AB/ThermoFischer is a commercial partner. The project follows the rules for IMI projects.

Thomas Schlederer, Linda Mathsson, Per Matsson, and Mats Nystrand are employees of Phadia AB/ThermoFischer.

Lars Klareskog, Vivianne Malmström, and Rikard Holmdahl are co-founders of a company, Curara AB, which collaborates with Phadia/ThermoFischer concerning certain technical aspects of the multiplex assay. This development is done with partial support from an ERC Proof of Concept Grant (pRActice) to LK.

Rikard Holmdahl is the inventor of patent US7/148,020B2, describing the diagnostic use of the C1 epitope.

Karin Lundberg is co-inventor of patent US12/524,465, describing the diagnostic use of the CEP-1 epitope.

As co-inventors of international patents about ACPA held by BioMérieux Cy and licensed to Eurodiagnostica Cy and Axis-Shield Cy, Leonor Nogueira and Guy Serre receive parts of the royalties paid to the Toulouse III University.

Johan Rönnelid has received remuneration for lectures and for the salary for Linda Mathsson from Phadia AB during the time she was employed in his laboratory. He has also obtained reagents from Phadia AB for the investigation on other rheumatoid arthris cohorts, as well as partial allowance for costs for attending congresses, in accordance with the rules established between Läkemedelsindustriföreningen (the trade association for the research-based pharmaceutical industry in Sweden) and the public Swedish health care system.

The other authors declare that they have no competing interests.

## Authors' contributions

MH planned the study and performed laboratory work with the ISAC chip and validation ELISAs. LM planned the study and performed laboratory work with the ISAC chip. TS performed the spotting of ISAC chips. LI established and performed validation ELISAs. PM planned the study. LN provided fibrinogen peptides and performed validation ELISAs. PJJ provided fibrinogen peptides. KL provided α-enolase peptide and participated in ELISA validation. VM participated in ELISA validation. GS provided fibrinogen peptides. RH provided collagen peptides. MN planned the study and participated in the laboratory work. LK planned the study. JR planned the study, performed statistical analyses, and drafted the manuscript. All authors read, commented on, and approved the final manuscript.
